# 6D Object Localization in Car-Assembly Industrial Environment

**DOI:** 10.3390/jimaging9030072

**Published:** 2023-03-20

**Authors:** Alexandra Papadaki, Maria Pateraki

**Affiliations:** 1 School of Rural Surveying and Geoinformatics Engineering, National Technical University of Athens, GR-15780 Athens, Greece; apapadaki@mail.ntua.gr; 2 Institute of Communication and Computer Systems (ICCS), National Technical University of Athens, GR-15773 Athens, Greece; 3 Institute of Computer Science, Foundation for Research and Technology-Hellas, GR-70013 Heraklion, Greece

**Keywords:** object 6D pose estimation, object localization, industrial robotic applications, challenging object characteristics, complex scenes, machine learning

## Abstract

In this work, a visual object detection and localization workflow integrated into a robotic platform is presented for the 6D pose estimation of objects with challenging characteristics in terms of weak texture, surface properties and symmetries. The workflow is used as part of a module for object pose estimation deployed to a mobile robotic platform that exploits the Robot Operating System (ROS) as middleware. The objects of interest aim to support robot grasping in the context of human–robot collaboration during car door assembly in industrial manufacturing environments. In addition to the special object properties, these environments are inherently characterised by cluttered background and unfavorable illumination conditions. For the purpose of this specific application, two different datasets were collected and annotated for training a learning-based method that extracts the object pose from a single frame. The first dataset was acquired in controlled laboratory conditions and the second in the actual indoor industrial environment. Different models were trained based on the individual datasets and a combination of them were further evaluated in a number of test sequences from the actual industrial environment. The qualitative and quantitative results demonstrate the potential of the presented method in relevant industrial applications.

## 1. Introduction

Object localization in robotic applications aims to support the identification of specific objects and the estimation of their 6D pose (rotation and translation in the camera coordinate system) for object-grasping and manipulation by the robot, extending to applications for human–robot collaboration. Commonly, objects with known 3D object models with annotated grasping points are used.

The main challenges in the aforementioned applications regard the nature of the industrial objects, the complexity and uncontrollability of the environment conditions and the requirements for accuracy and real-time processing. In industrial environments, as also in the case of the car assembly line, the objects of interest are usually tools or manufacturing components with challenging geometric and surface characteristics, such as symmetries, reflective surfaces and with weak or no texture, featuring also similarities to other objects in the scene (e.g., different types of screwdrivers). Furthermore, in these environments usually unfavorable illumination conditions are met, while different static, moving, overlapping objects and assembly line components contribute to significant amounts of background clutter and occlusions. One has also to consider the deployment on robotic platforms with hardware constraints in terms of size, memory and battery, enabling in parallel successful, accurate and real-time object localization.

The developed workflow aims to support robot grasping, as part of human–robot collaboration during car-door assembly tasks. In this scenario the robot has to detect specific objects, to grasp and hand over them to the worker, ensuring fluent human–robot interaction and unobstructed assembly flow. In fact, the case of a specific car assembly line workflow is examined that considers all the aforementioned challenges regarding both the characteristics of the industrial objects and the manufacturing context.

The main contribution of the presented work is the application of 6D object localization in a real industrial environment, exploiting objects commonly used in car-assembly tasks, combining deep learning methods for pose estimation, data augmentation for training models, based on different datasets relevant to the application domain captured in a systematic manner, and validating results on test sequences from the actual manufacturing environment. Results indicate that object and scene challenges are successfully handled with the trained models, achieving average recall scores that exceed 94% based on different evaluation metrics and time performance of less than 0.4 s per image.

**Figure 1:**
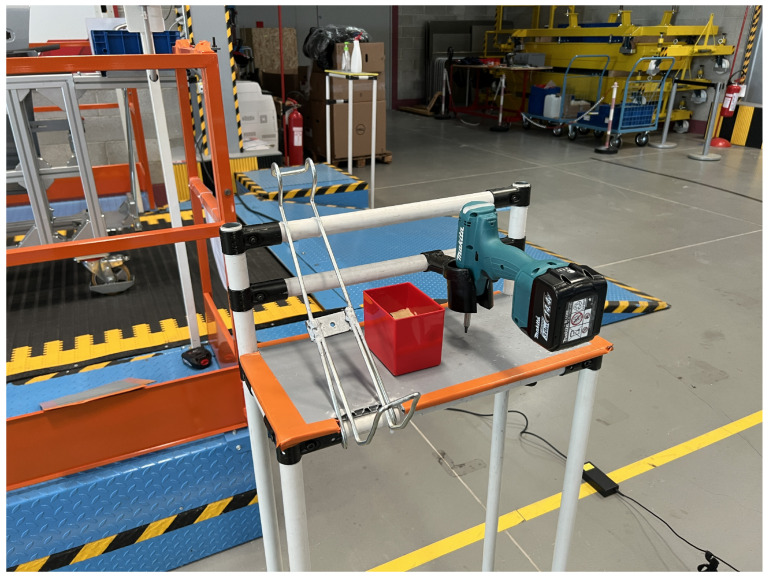
View of the assembly line depicting the screwdriver placement.

**Figure 2:**
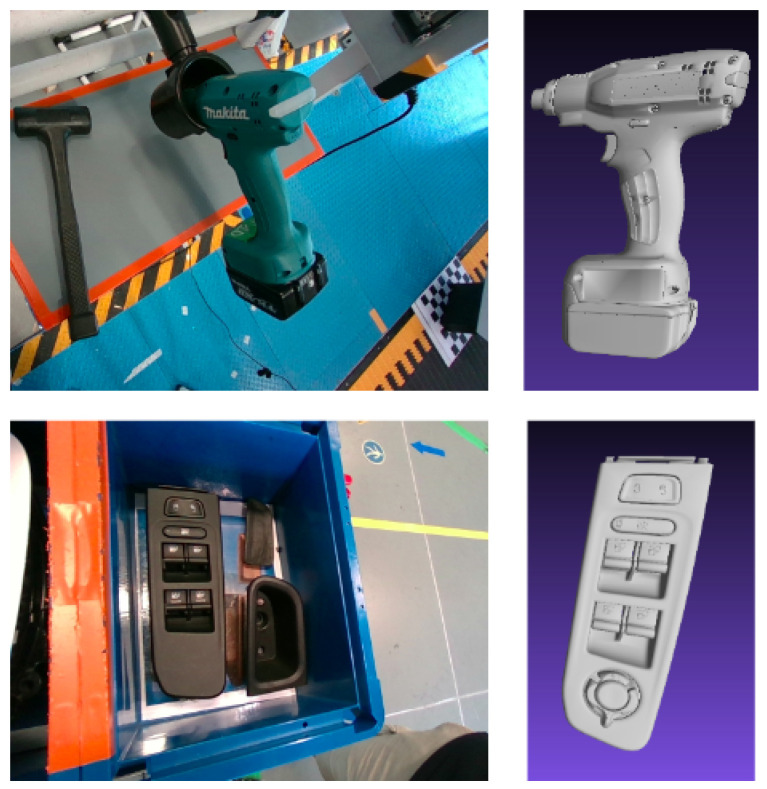
Objects investigated in the examined application, a screwdriver (row 1) and a black window control panel (row 2). The first column presents sample images of the objects in the industrial environment, while the second column presents the respective 3D object models.

The developed methodology uses as input a single RGB image, captured by a camera mounted on the robot arm and queries specific objects. The EPOS method [1] is used as the baseline deep learning method to predict the object pose based on different collected and annotated training datasets. Initially, training data were captured for the objects of interest in laboratory conditions in a systematic manner. The objects were placed on a flat surface and the training images were covering a full view sphere, allowing the model to be trained on a large number of possible object placements and camera viewpoints. On the contrary, in the case of real industrial environments, as the one of the examined car assembly line (see example scene in Figure 1), certain objects are placed in specific positions, such as hangers, boxes, and trolleys. This setup restricts the number of alternative locations and orientation of objects in the shop floor, therefore reducing the viewpoints and object poses to be handled by the method. On the other hand, the assembly line environment is very complex, with unfavorable illumination conditions and different objects, that is, car components, moving objects, and so forth, that are depicted as cluttered background in the input images. To further address these challenging scene conditions, additional data were captured in the actual industrial manufacturing environment and were used for model training. For the purpose of this study two objects used in the car assembly line were considered, namely the screwdriver and the black window control panel, as shown in Figure 2.

## 2. Related Work

6D object localization or else object pose estimation deals with the problem of estimating the location of known objects present in a scene, based on computer vision methods. It calculates the transformation from the coordinate system of the object in the scene to the coordinate system of the camera. This transformation is defined as a translation and a rotation matrix.

6D pose estimation methods are extensively reviewed in several works, for example, [2,3,4,5,6,7,8]. These usually categorize object localization according to the prior information, the number of views and the type of computer vision methods being used. Object detection and localization methods differ based on whether they use 2D or 3D information derived by different sensors [1,5,9], single or multiple views [10], classic or deep learning methods [1,3,5] and correspondence, template or voting based approaches [3]. More recent works exploit deep learning methods, the development of which has given the 6D object pose estimation a great boost [11]. In parallel, source code implementations of relevant research developments for 6D object pose estimation are publicly available [12], as well as a number of annotated datasets [13].

One of the most important open issues in the literature regarding the 6D object localization is handling objects with weak texture, symmetries, and reflective surfaces as well as challenging scenes with occlusions and clutter. These cases are considered in the current work. Three methods that featured in the 2020 BOP challenge [13], CosyPose [10], EPOS [1] and Pix2Pose [14] aim to address the above challenges and are therefore relevant to the application domain of this work. CosyPose [10] is a single-RGB 6D pose estimation method, that handles symmetric and occluded objects, using a render-and-compare DNN method inspired by DeepIM [15] (using EfficientNet-B3 [16] instead of FlowNet [17] commonly used in DeepIM). Initially, it detects all known objects in the image and then for each object it assumes a canonical pose creating a corresponding rendered image. By comparing the rendered image to the input, it estimates a coarse pose, which is then refined using a similar iterative refiner DNN network. The pose hypothesis can be further optimized by matching pose hypotheses across the different views and applying global scene refinement. EPOS [1] is a single-RGB, correspondence-based method that defines object–surface fragments to handle both global and partial symmetries. It predicts pixel-fragment correspondences, using an encoder–decoder network. Finally, EPOS overcomes the many-to-many correspondences issue and recovers all the object instances using a PnP algorithm [18] within a RANSAC framework [19]. Pix2Pose [14] also handles occlusions, using only RGB images and models without texture. However, it uses one DNN model for each object. It performs pixel-wise prediction of the 2D–3D correspondences without having to define specific keypoints and also relies on segmentation instead of detection. It predicts the corresponding 3D coordinates for each densely sampled image pixel, using an auto-encoder architecture, and then uses the PnP algorithm [18] with RANSAC [19] iterations to directly calculate the object poses. Pix2Pose also manages to handle global object symmetries using a novel transformer loss function, failing to successfully handle all cases of partial object symmetries though.

Further to these three methods, DOPE++ [20] used RGBD images and applied a random mask local processing method and a multiscale fusion pose estimation module to cope with occlusions and scale differences for weakly textured objects. Depth information was also taken into account in other recent works. PVN3D [21] used an RGBD input image and performs direct regression using a deep Hough voting network followed by a least-squares fitting and FFB6D [22] used a Full Flow Bidirectional fusion network that are both fed with a single RGBD image, combining the appearance information from the RGB image and the geometric information from the depth image. On the contrary, FS6D [23] also uses a single RGBD input image to combine both appearance and geometric information, but tackles the problem of multiple object instances in the scene and also the restriction of the known object 3D model, using a few-shot 6D pose estimation method. DGECN [24] takes advantage of the geometric features in 3D space and proposes a depth-guided edge convolutional network. In the current work, depth information was also available since an active stereoscopic RGBD camera was used. However, the camera has a minimum depth distance of around 52 cm, thus for operating distances smaller than 52 cm the depth information would be of degraded accuracy. Though as shorter operating distances may encounter in our application paradigm we decided to rely only on RGB data.

Most of the recent works [21,22] manage to achieve very high scores in recall accuracy on benchmark datasets. However, the majority of benchmark datasets used for object detection and localization, analysed also in Section 3.1, introduce some restrictions, avoiding to handle objects with symmetries, and reflective surfaces and obtaining both training and testing data from the same object space. These assumptions, though, limit the applicability and robustness of object detection and localization, especially in real-time robotic applications in industrial environments, where objects with the aforementioned characteristics are commonly met.

The latest works try to tackle those issues using different approaches. To lift the restriction of using training and testing data from the same object space, a category-level pose estimation task that generalizes to new objects but still keeps the restriction of the predefined object categories has been recently proposed [25]. Regarding the required training data, data augmentation has been widely used for increasing the model accuracy, while requiring a smaller amount of training data. However, in most cases, simpler methods of data augmentation such as geometric and intensity transformations (rotation, translation, guttering, blurring, and so forth) [1,23] are used. In most cases the augmented images are prepared offline since generating object textures, materials and photorealistic rendering require considerable human effort, processing time, and computational cost. This offline process, however, adds a time-consuming step before the training of the model, and the requirement for extra storage space. To overcome the time-consuming and non-scalable annotation of real data and the lack of realism of the synthetic data, recent works exploit NeRFs and the first-reconstruct-then-regress idea [26] to produce annotated data. The aforementioned methods and frameworks achieve very accurate results primarily in offline applications and on benchmark datasets, that usually depict household or simpler industrial objects on simple backgrounds. To the best of the author’s knowledge, none of them has been applied to real world industrial robotic applications.

## 3. Methodology

As mentioned above, the main goal of the developed methodology is to detect and estimate the 6D pose (rotation and translation) in the camera coordinate system of specific known industrial tools and objects in real time. The subsections below analyse the objects and environmental conditions that are handled, the pose estimation method and the evaluation metrics that are used, the processing applied on the 3D object models and the deployment of the methodology on the robotic platform.

### 3.1. Challenging Objects and Environmental Conditions

The industrial objects and tools of interest feature challenging properties such as symmetries and surfaces that are reflective, absorbing (black) or textureless.

**Figure 3:**
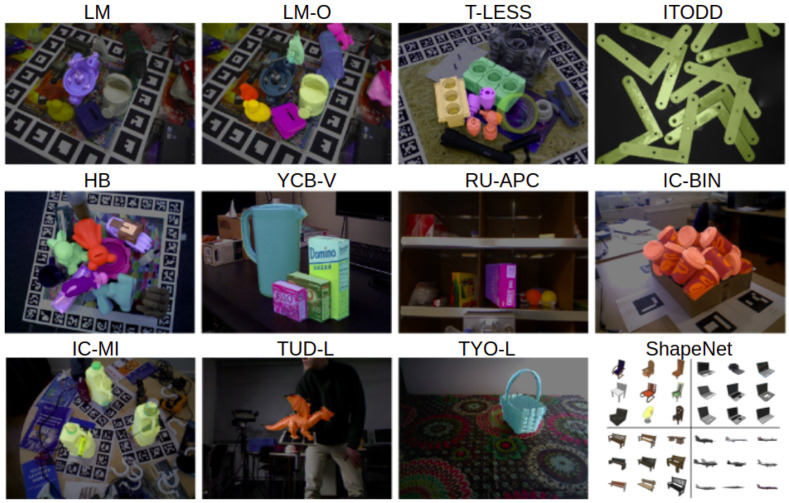
Benchmark datasets widely used for object detection and 6D pose estimation: LM [27], LM-O [28], T-LESS [33], ITODD [34], HB [29], YCB-V [32], RU-APC [30], IC-Bin the Ref. [35], IC-MI [31], TUD-L and TYO-L [38] and ShapeNet [36].

A large number of available benchmark datasets are widely used for object detection and localization. These datasets, as shown in Figure 3, depict objects of varying sizes and characteristics, either isolated or as part of complex scenes with multiple object instances. LM [27], LM-O [28], HB [29], RU-APC [30], IC-MI [31] and YCB-V [32] contain objects of everyday life, depicted in complex scenes. T-LESS [33] depicts industrial objects with symmetries, occlusions, and weak texture, while ITODD [34] and IC-Bin the Ref. [35] depict multiple instances of identical objects, simulating the bin-picking problem. ITODD [34] represents one of the most hard-to-solve cases of identical overlapping, reflective metallic objects under imperfect illumination conditions. Most of the developed methods in the literature have been tested on such benchmark datasets, while some of them have also created and introduced new synthetic photorealistic datasets [20,23,36,37].

However, the current application scenario contains car components and tools of a car assembly line, namely the screwdriver and the black window control panel, shown in Figure 2, and none of the available benchmark datasets was representative enough to serve the needs of our application. For that reason, new training data with the objects of interest were acquired for the model training. Starting with the two aforementioned objects and establishing the developed methodology, additional industrial objects could be incorporated.

Apart from the characteristics of the specific objects, additional context-related challenges are encountered, thus occlusions, complex backgrounds and clutter, variable illumination and objects placed in several different locations (boxes, dollies, trolleys, and so forth) (Figure 1).

### 3.2. Object Pose Estimation

To establish a proper object detection and localization pipeline, a thorough investigation and testing of the most relevant state-of-the-art methods mentioned in Section 2, based on Deep Learning was performed on benchmark data. Cosypose [10], EPOS [1] and Pix2Pose [14] were tested initially on LM-O [28] and T-LESS [33] datasets, as these were considered most relevant, due to the variety of challenging objects with occlusions and clutter. The aforementioned methods were able to handle objects with special geometric and textural characteristics and complex scene conditions, it was decided to further exploit the EPOS method [1], due to its time efficiency during inference and its agility to handle multiple objects with a single Deep Neural Network (DNN) [13].

The EPOS algorithm [1] accepts as input the RGB information from the images, that are in the context of this application captured by the camera in real-time. The additional depth information, can be also exploited to improve the accuracy of the required rendering of the object models that are already available. It should be highlighted though that such additional depth information was not used in the current work, since accurate depth information would not be obtained for small distances between the object and the camera, that is, less than 50 cm. For representing an object, a set of compact surface fragments are used, enabling EPOS to deal with symmetries. During inference, for each image pixel the 3D locations of possibly multiple fragments are predicted, allowing to capture object symmetries. Additionally, many-to-many 2D to 3D correspondences are established by linking pixels with the predicted 3D locations, and a robust and efficient variant of the PnP-RANSAC algorithm [18,19] is used to estimate the 6D poses [1].

Initially, a regressor associates each of the surface fragments of the object to predict the corresponding 3D location expressed in 3D fragment coordinates. Consequently, a single deep convolutional neural network (DNN) with a DeepLabv3+ [39] encoder–decoder is adopted to densely predict (1) the probability of each object’s presence, (2) the probability of the fragments given the object’s presence, and (3) the precise 3D location on each fragment in 3D fragment coordinates. For training the network, a per-pixel annotation in the form of an object label, a fragment label, and 3D fragment coordinates is provided. Additionally, the average softmax cross entropy loss and Huber loss [40] over all pixels are calculated, while the necessary vectors for calculating losses are obtained by rendering the 3D object models in the ground truth pose with a custom OpenGL shader.

To estimate the 6D poses of multiple instances of an object from the 2D–3D correspondences, a robust and efficient variant of the PnP-RANSAC algorithm [18,19] is used, integrated into the Progressive-X scheme [41], an efficient multi-model fitting algorithm. The 6D pose hypotheses are then proposed by GC-RANSAC [42], a locally optimized RANSAC and the pose is estimated by the P3P solver [43]. Then, the pose is refined from all inliers by the EPnP solver [18] followed by Levenberg–Marquardt optimization [44]. Finally, for the verification of the 6D pose, its quality is calculated, considering only the most accurate correspondence as only up to one correspondence may be compatible with the hypothesis [1]. Pixels outside the visibility masks of the objects are considered to be the background. While the masks in the original approach are calculated as in the Refs. [1,45], an improvement in that direction was implemented in the context of this work, by exploiting a U-Net [46] based approach for the laboratory data, for which the initial mask precision was not adequate (see Section 4.1.1).

### 3.3. Evaluation Metrics

The accuracy and precision of the 6D object pose estimation is evaluated through the comparison between the predicted and the known ground truth pose of the depicted object. A well-established strategy from literature [13] is exploited to evaluate the 6D object pose estimation based on several metrics. The most widely used metrics are: (i) Average Distance for distinguishable (ADD or ADD(-S)), symmetric distinguishable (ADD-S) and indistinguishable (ADI) objects, which calculates the average distance between the vertices in the 3D space. However, this metric depends highly on the object model geometry and the surface sampling density, because it takes into account the average distance; (ii) Visible Surface Discrepancy (VSD), which defines the object location using distance maps between the input and rendered image, while being invariant to symmetries and encountering only the visible object parts; and (iii) Maximum Symmetry-Aware Surface Distance (MSSD) that computes the maximum distance and is independent of the object geometry. This metric is critical for applications such as the examined one, since it indicates the chance of a successful object grasp [13]; (iv) Maximum Symmetry-Aware Projection Distance (MSPD) that is similar to MSSD but for the 2D space, leaving out the Z misalignment. These metrics reflect each a different perspective of object pose estimation and are usually used in a combination to extract the final estimation accuracy [13]. This evaluation strategy was also followed in the presented methodology. The recall rates for the several metrics were calculated for different correctness threshold settings according to the object model diameter or the image width. In fact, thresholds were ranging from 10% to 50% of the object diameter with a step of 5% for the VSD, from 5% to 50% of the object diameter with a step of 5% for the MSSD, from 5r to 50r with a step of 5r, where r = w/640 and w being the image width in pixels for the MSPD and less than 10% of the object diameter for the ADD metric.

### 3.4. Object Models

A collection of 3D mesh models of the known objects was created to be used as prior knowledge, complying with the BOP dataset format [13]. The initial CAD object models were provided by experts in *jt* format. The models were transformed into *ply* format and processed to include surface normals at the model vertices, calculated using MeshLab [47] as the angle-weighted sum of face normals incident to a vertex [1,14]. Views of the 3D models of the examined objects are shown in the second column of Figure 2.

### 3.5. On-Robot Application/Integration

The developed methodology requires that it is fully integrated to a robotic platform, supporting real-time performance. For this purpose, the on-robot hardware was selected and the implementations were developed so that they comply with the memory and computational restrictions of a moving robot.

The mobile robotic platform carries an on-robot camera (Intel RealSense D455 active stereoscopic camera [48]), featuring compact size and global shutter, that captures the input data on demand and in real time. The camera projects infrared light and manages to provide high-quality depth information even under unfavorable lighting conditions, while it is less affected by interference signals by other cameras, such as in the case of the time-of-flight active cameras. The only limitation is the minimum depth distance of around 52 cm.

**Figure 4:**
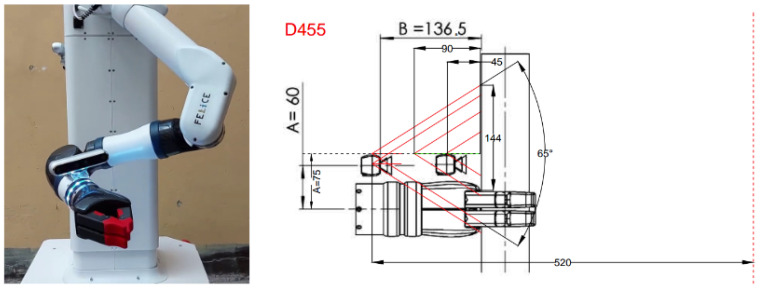
On-robot camera. The (**left**) image depicts the Intel RealSense D455 camera mounted on the robot arm. The (**right**) image illustrates the investigation of the optimal placement of the Intel Realsense D455 camera on the robot arm. All the dimensions shown are in mm. The dashed red line on the right represents an object to be localized.

**Figure 5:**
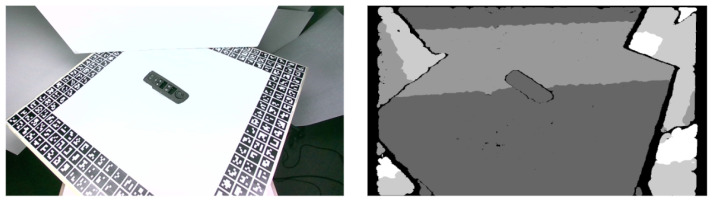
RGB (**left**) and depth (**right**) images from a Intel RealSense D455 camera, from the created laboratory dataset.

The camera was mounted on the robot wrist, as shown in the left image in Figure 4, and placed on an adjustable holder that provided the optimal camera positioning for the object localization. The exact position and orientation of the camera were decided according to the field of view of the camera, its minimum depth distance for representative depth information, as well as the requirements for safe object handling and grasping, given that the used image resolution was decided to be the one that provides the wider possible field-of-view, namely, 1280 × 720 pixels. The right image in Figure 4 presents the investigated alternatives for the optimal camera viewpoint and placement aiming to maximize the imaged area with minimal occlusions from the robot wrist and ensure safe object-grasping without risking to damage the camera. For that reason, it was decided to place the Intel Realsense D455 camera on an adjustable base that slightly raises the camera above the arm (75 mm from the center of the arm shown as distance A in the right image in Figure 4) and allows it to move 90–136 mm from the beginning of the robot fingers (depicted as distance B in the right image in Figure 4). For these calculations, a minimum camera-to-object distance of around 52 cm was assumed to also comply with the minimum depth range of the Realsense D455. The on-robot camera was calibrated in order to calculate its intrinsic parameters, using the Intel® RealSense™ D400 Series Dynamic Calibration Tool. Sample RGB and depth images captured by the Intel RealSense D455 camera are shown in Figure 5.

ROS was used as the middleware for communication of the object detection and localization module with the on-robot camera and other robot modules.

- **ROS integration of RealSense2:** The integration of the used RealSense camera was achieved through the wrapper provided by Intel. A ROS node was created to receive aligned images and camera calibration parameters from the RealSense camera, that would be further used as inputs for the object pose estimation.
- **Detection and Localization node:** The functionality of the object localization was integrated into ROS using a ROS package. This package contains a node that receives the input data from the RealSense camera and runs the object detection and localization as an on-demand ROS service, requested to localize an object, and the results are further communicated through a ROS message.

## 4. Experiments and Results

The EPOS framework was initially tested using available models on benchmark data, though these were considered inadequate to represent the actual objects and the environment conditions. Therefore, new datasets with the objects of interest were created for training and validation. A laboratory dataset was acquired under controlled conditions, as well as an additional dataset from the indoor environment of a real car assembly line and with the objects placed in realistic positions and poses in the production workstations.

**Figure 6:**
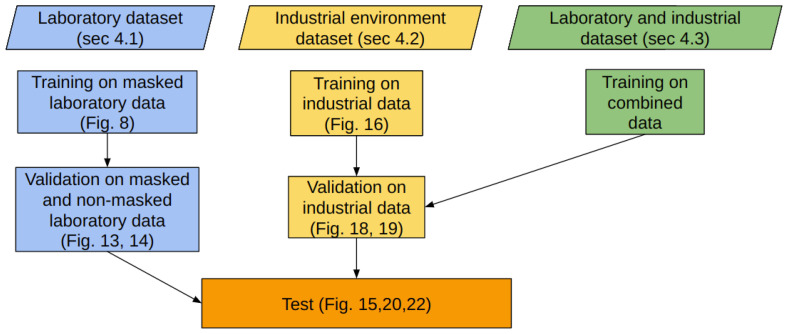
Workflow of the performed experiments, including the training, validation, and testing steps for the three models trained in the laboratory (**blue**), in the real environment (**yellow**) and in a combination of the laboratory and real environment (**green**) data. In the parentheses the respective sections and figures are cited.

The trained models based on these newly collected datasets were tested on a number of test sequences from the actual industrial environment. Figure 6 presents the experimentation procedure that was followed in the current work, including the training, validation and testing steps for the different types of datasets. All trained models are thoroughly analyzed in the respective sections below.

### 4.1. Model Trained on Laboratory Data

#### 4.1.1. Datasets

The laboratory dataset is unique as it depicts the objects of interest under controlled conditions and in a systematic manner, using the Realsense D455 sensor. The calculated ground truth poses and the 3D models of the objects are also included.

**Figure 7:**
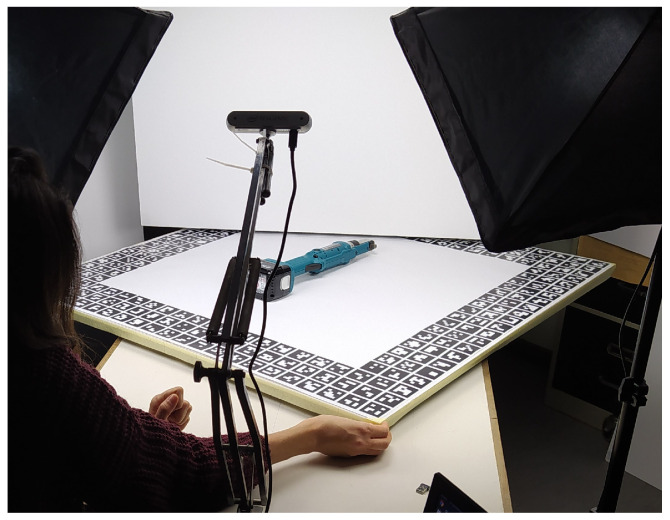
Setup prepared for capturing training and validation images in the laboratory. Objects are placed on a turnable table and the camera is mounted on an adjustable arm.

In order to capture the training and validation RGBD images for this dataset, a dedicated setup as shown in Figure 7 was prepared. In this setup, the objects were placed on a turnable surface and the camera was placed on an adjustable arm that allows us to capture images from different angles and tilts. A whiteboard with markers around the border area (specifically AruCo [49]) was placed on the turnable table, to later facilitate the estimation of the ground truth poses. The objects were placed approximately at the center of this board, to ensure a uniform background for all viewpoints. The images were captured from a depth range of 0.53–0.80 cm, adhering to the minimum depth range of the Intel RealSense D455 camera, in order to create a generic benchmark dataset with meaningful RGB as well as depth information.

**Figure 8:**
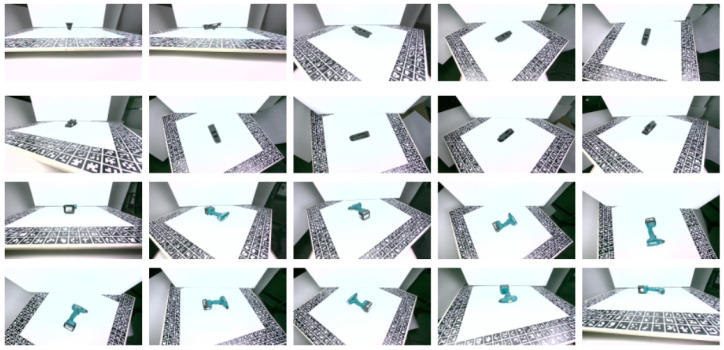
Sample images from the dataset created in the laboratory for the window control panel (rows 1 and 2) and the screwdriver (rows 3 and 4).

The images were acquired in a systematic manner, following a dense spherical net of viewpoints that ensured a dense sampling of all the possible poses of the depicted object, similarly to the T-LESS dataset [33]. The acquisition covered all viewpoints with elevation from −85 degrees to 85 degrees with a 10 degrees step and also 360 degrees azimuth range with a step of 5 degrees. To cover the full elevation range, the object had to be flipped. Following this process, 1296 images were captured for each object, 75% of which were used as training and 25% as validation data. Figure 8 depicts sample training images from the laboratory dataset.

To remove background clutter, the images were masked, following the standard EPOS process [45], though it was noticed that the derived masks were not always accurate enough. For that purpose, several state-of-the-art Fully Convolutional Networks (FCNs) for image semantic segmentation, such as FCN-ResNet50, FCN-ResNet101 [50], DeepLab + ResNet50, DeepLab + ResNet101 [51], SegNet [52], and U-Net [46] were trained and tested on the dataset. The resulting masks were compared to the ones derived from the EPOS standard masking method. The U-Net method was finally decided upon to replace the standard masking approach used by EPOS, since it provided the most precise and complete masks.

**Figure 9:**
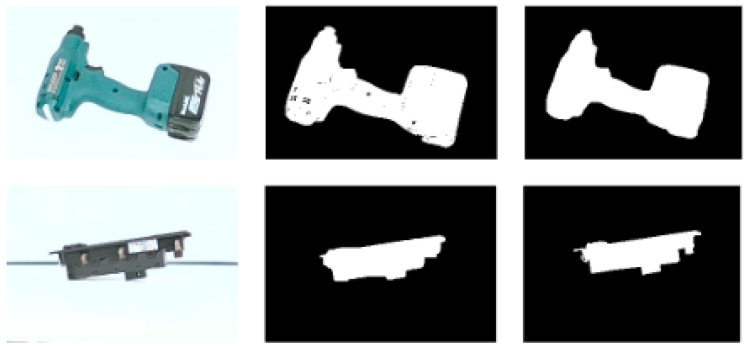
Closeups of examples of masks calculated by the standard EPOS masking method [45] (**middle** column) and U-Net (**right** column) along with the original image (**left** column).

**Figure 10:**
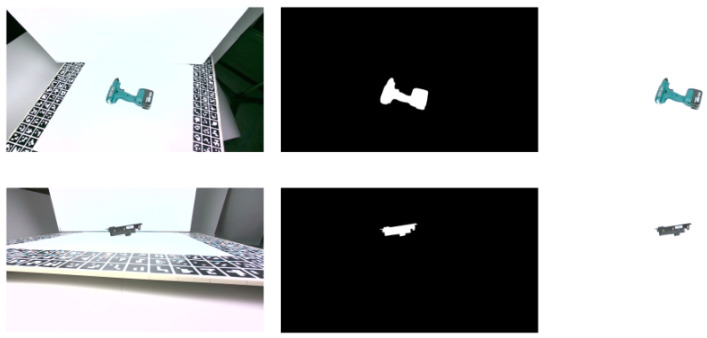
Examples of initially captured (**left** column) and final training (**right** column) images, masked using the U-Net masks (**middle** column).

Figure 9 below depicts indicative closeups of the captured images with background clutter (left column), the masks computed by the standard EPOS masking method (middle column) and the masks computed by U-Net (right column). It can be noticed that in the example of the first row, certain details are missing from the mask created using EPOS, with apparent holes in the created mask, while U-Net has managed to mask only the background. On the contrary, in the example of the second row, the mask created by the standard EPOS method is more generalized and less accurate than the one created by U-Net. Examples of the finally masked images to be used for the model training are presented in the last column of Figure 10.

The created masked images were used as training and validation data, while the initially captured non-masked images were only used for validation.

To obtain the ground truth object poses that were required for training, the pose of the depicted object with respect to the camera coordinate system had to be calculated for each image. For that purpose, the transformation of the object pose from the 3D model system to the world (AruCo) coordinate system was calculated, encountering the world to camera transformation that was computed for each image using the AruCo markers and the average model to camera transformation, computed for a small and representative number of images. The calculated object pose in the world coordinate system was finally transformed back to the camera coordinate system for each image, based on the transformation defined by the AruCo board. The aforementioned transformations were calculated by solving the 2D–3D correspondences of the depicted object with respect to its 3D model or the world coordinate system (AruCo marker system), respectively, using the Posest library [53,54].

**Figure 11:**
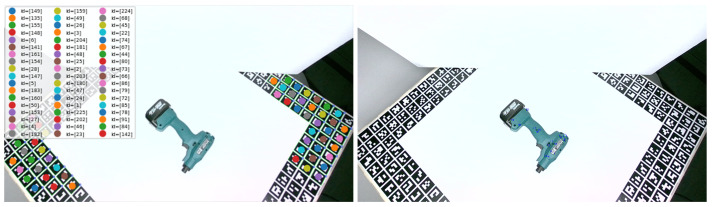
Automatically detected AruCo markers (**left**) and manually selected object points (**right**) on the training images, used to calculate the ground truth object pose.

The required 3D coordinates of the AruCo markers were manually measured, while the corresponding 2D image points were automatically detected on all images, as shown on the left image of Figure 11. The 3D points on the objects were measured on the 3D object models in Meshlab, while the corresponding 2D image points were manually selected on a small but representative number of images, as shown on the right image of Figure 11.

#### 4.1.2. Training, Validation and Testing Results

For optimizing the object detection and localization network for the laboratory training dataset, a DeepLabv3+ encoder–decoder network [39] with an Xception-65 [55] network as the backbone was used. The backbone network was pre-trained on the ImageNet [56] and COCO datasets [57]. Consequently, the object detection and localization model was trained on 972 images per object, for both objects shown in Figure 2.

**Figure 12:**
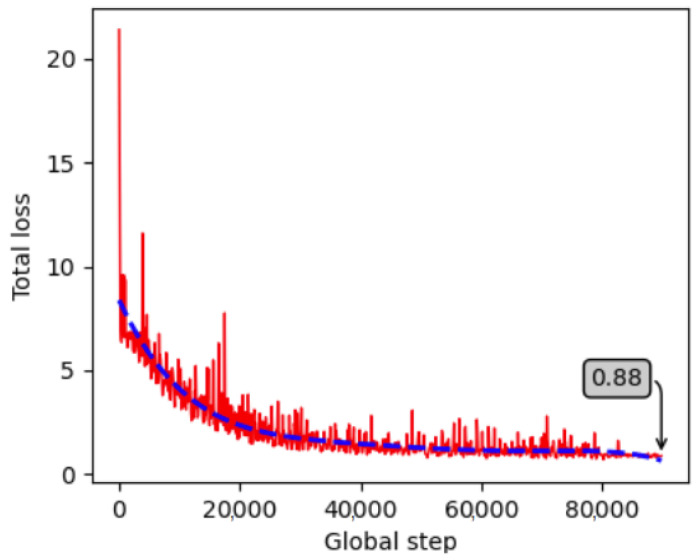
Progress of the training loss for the created laboratory dataset.

After a thorough investigation of the model architecture, the model was finally trained for 90,000 steps, starting from a low learning rate of 0.00005 with a learning power of 0.7, to avoid overfitting. Each object was fragmented into 64 surface fragments. Both objects were trained in a single model and training was completed, achieving 0.88 loss. The training progress is visualized in Figure 12, indicating that the loss is minimized and stabilized. To prevent overfitting, the images were augmented during training by randomly adjusting brightness, contrast, hue, and saturation, and by applying random Gaussian noise and blur, similarly to the Ref. [58]. The model was then validated on 324 masked and 324 non-masked unseen images per object. The rest of the section presents the quantitative and qualitative results.

**Figure 13:**
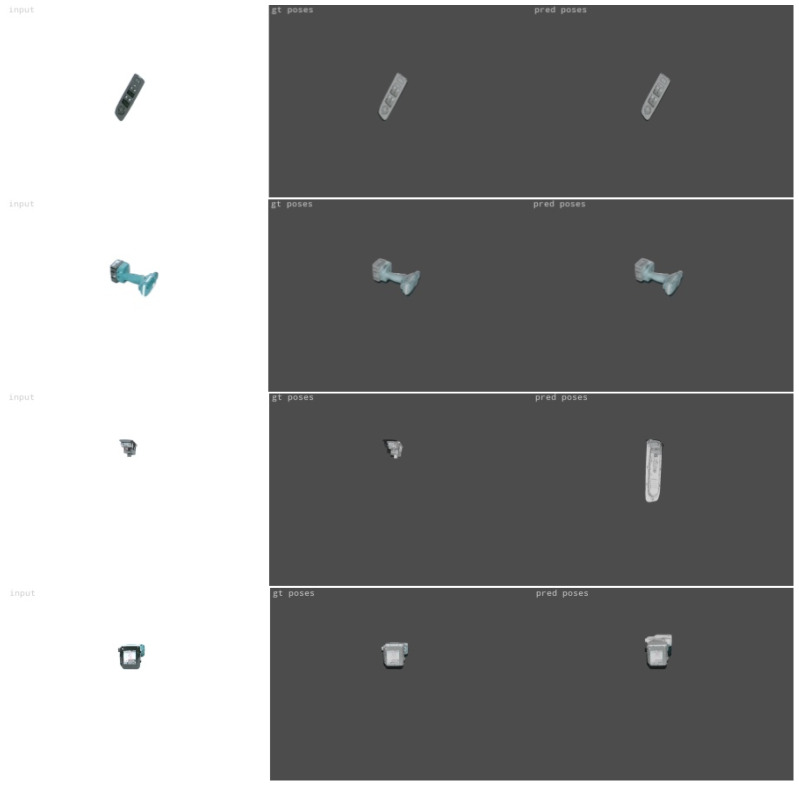
Examples of precise predictions (first and second rows) and of incorrect predictions (third and fourth rows) on masked images. The first column presents the input validation image, the second column the ground truth and the third one the estimated object pose.

**Figure 14:**
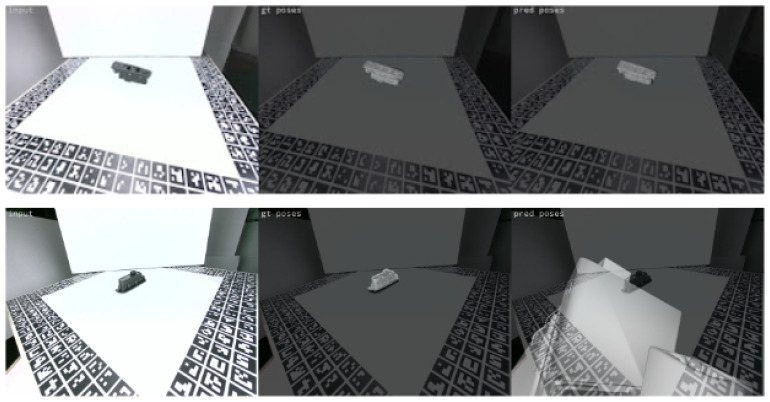
Examples of correct (row 1) and incorrect (row 2) predictions on non-masked validation images. The first column presents the input validation image, the second column the ground truth and the third one the estimated object pose.

Figure 13 presents indicative results for the masked images. The first two rows show examples of very high-precision predictions, while the last two rows present rare cases of incorrect object pose prediction that are mainly caused by the very low elevation imaging angle that depicts the objects in a very hard-to-interpret pose. Figure 14 depicts sample results for the case of the non-masked validation images. The first row presents an example where the object is accurately detected and localized, regardless of the cluttered background. However, in some cases, the non-uniform background led to incorrect predictions, especially at unfavorable low-elevation imaging angles, such as in the case presented in the second row of Figure 14.

Except for the visual qualitative evaluation, the object detection and localization results were also evaluated quantitatively based on the state-of-the-art evaluation measures that were presented in Section 3.3.

Table 1 presents the average recalls for the MSSD, MSPD, ADD, and VSD metrics, as well as the average of the average recalls that is more commonly met in the literature. The first row shows the results for the masked validation images, whereas the second one shows the results for the initially captured non-masked images. The recall scores for the case of the masked images are very high with an average of MSSD, MSPD and VSD metrics of up to 97.8%. On the other hand, the average results for the non-masked images are up to 50.9%, close to the results on benchmark datasets, but still much lower than the masked images. This reduced average recall reveals the need for further training of the model on more realistic data in more complex scenes, which will be achieved by training on real data captured in the industrial environment (see Section 4.2) and maybe also augmented data derived from them.

**Figure 15:**
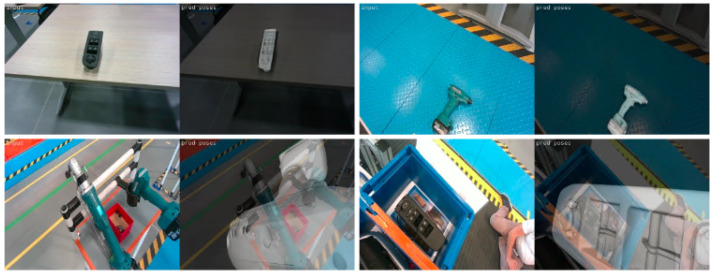
Test results for the model trained on laboratory data. The first row illustrates correct predictions in cases of homogeneous background, while the second row illustrates cases of incorrect predictions due to complex background.

The model trained on laboratory data was also tested on unseen real environment data of homogeneous and complex backgrounds. No ground truth poses were available for this dataset, however, the results were visually evaluated. Figure 15 presents such examples, where it can be observed that the model predicts with a pretty high accuracy the pose of the objects on a quite homogeneous background. However, as shown in the second row, it fails to deal with complex scenes. To improve the method robustness and enable it to handle cases of more complex scenes with cluttered backgrounds and occlusions, like the actual industrial environments, the model was also trained with data from the actual manufacturing assembly line environment (see Section 4.2 and Section 4.3).

The laboratory data could be used in the future to produce augmented data in a more intelligent way, in order to increase the amount of training data without requiring additional annotation effort, while at the same time enabling the application to be robust enough to perform well for a variety of complex environments.

### 4.2. Model Trained on Real Industrial Environment Data

#### 4.2.1. Datasets

In the previous section it was proven that the model trained on masked laboratory data delivers high accuracy in localizing the investigated objects in uniform and homogeneous backgrounds. However, the accuracy decreases dramatically when the background clutter and the complexity of the scene increases. To address this issue, the model was re-trained with real data from the assembly line environment, captured using the Realsense D455 sensor and calculating the ground truth poses.

**Figure 16:**
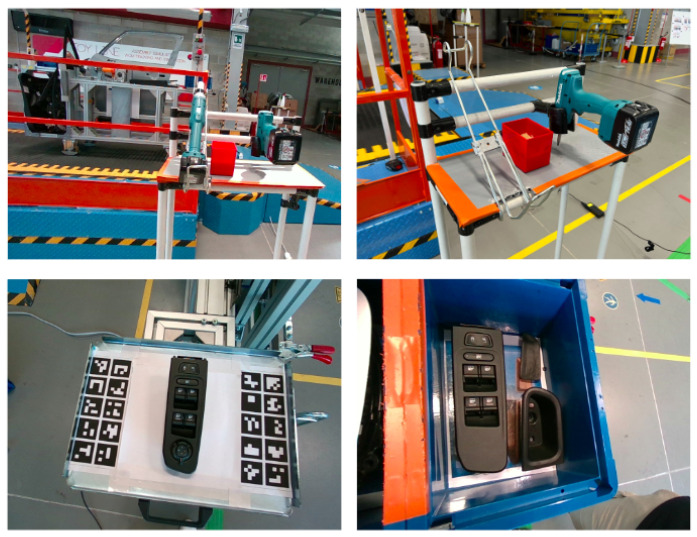
Sample training images from the real industrial environment dataset for the screwdriver (**top** row) and the window control panel (**bottom** row).

The training and validation RGBD images for this dataset did not require a special setup such as in the case of the laboratory dataset. In this case, the objects were placed in realistic positions in the car assembly line environment. The images were manually captured from a depth range of around 0.30–0.80 cm to comply with the safety regulations of the robot mobility with respect to possible collisions with the surrounding environment. The captured images covered a quarter-sphere net of viewpoints from many different angles and tilts, to ensure a dense sampling of all the possible viewpoints of the robot camera to the objects in their positions in the real environment. Furthermore, the dataset was captured in real conditions, depicting the real clutter and background noise in the car assembly line environment. Finally, 600 images were captured for each object, with 75% of them used as training and 25% as validation data. Sample training RGB images from the real environment dataset are presented in Figure 16.

In this case, the input training and validation images were not masked before feeding them to the network. Additionally, on the contrary to the laboratory data, no markers were needed since the ground truth object poses in the images were calculated using the 6D-PAT pose annotation tool [59].

#### 4.2.2. Training, Validation and Testing Results

Similarly to the model trained on the laboratory data, the optimization for training on the real environment data was achieved using a DeepLabv3+ encoder–decoder network [51] with an Xception-65 [55] network, pretrained on ImageNet [56] and the COCO dataset [57], as a backbone. The object detection and localization model for the case of the real data was trained on 450 images per object, for both the examined objects presented in Figure 2.

**Figure 17:**
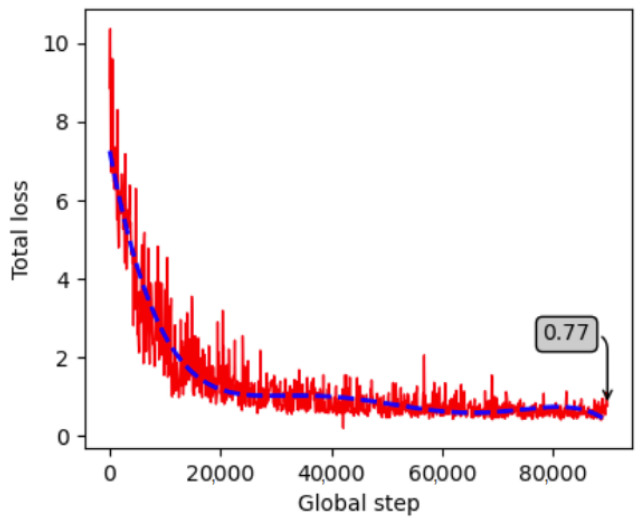
Progress of the training loss for the created dataset in the real industrial environment.

The most well-performing model architecture was found to be that of the 90,000 training steps, with an initial learning rate of 0.0001, a learning power of 0.9 and 64 fragments for each of the two examined objects. A final trained model for both objects was created, achieving a 0.77 training loss. Figure 17 depicts the training progress for this model, showing that at the end the loss is minimized and stable. During training, the training images were augmented similarly to the images for the model of the laboratory data (see Section 4.1.2), to avoid overfitting. The model validation was then performed on 150 unseen images for each object.

**Figure 18:**
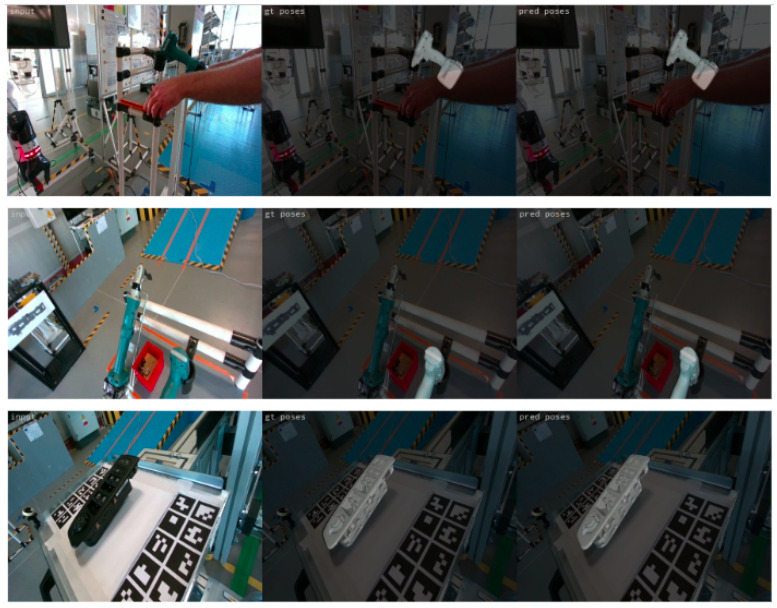

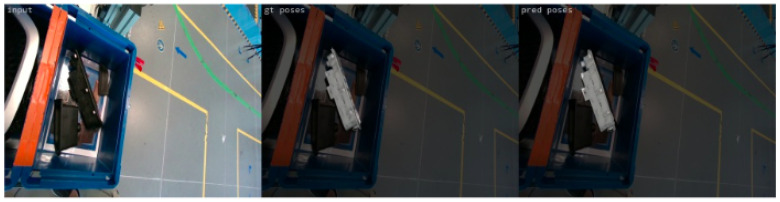
Examples of precise predictions on validation images from the real industrial environment. The first column presents the input validation image, the second column the ground truth, and the third one the estimated object pose.

Figure 18 provides some visualization of the ground truth and the estimated object pose for the validation data from the real environment. The first two rows present results for the screwdriver, while the next two present results for the window control panel. The method indicates superior performance even in cases of occluded and partially depicted objects, very complex and noisy backgrounds, and unfavourable conditions that even make the features of the objects very hard to distinguish with the naked eye, especially in the example in the last row of the black window control panel.

**Figure 19:**
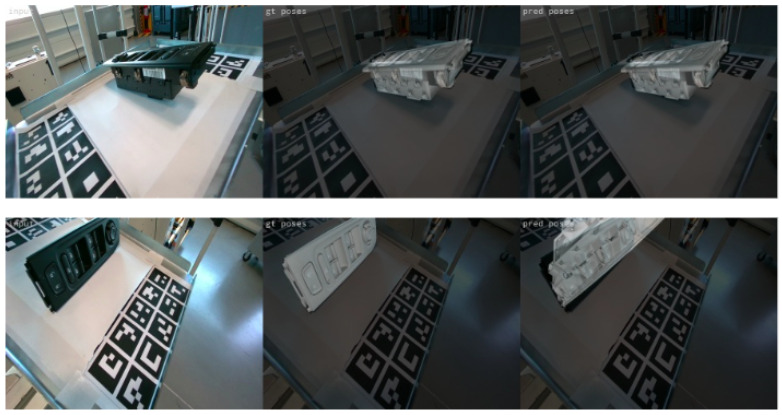
Examples of incorrect predictions on validation images from the real industrial environment. The first column presents the input validation image, the second column the ground truth, and the third one the estimated object pose.

Figure 19 illustrates the results of some of the very few cases of incorrect pose estimates on the validation data. These rare cases of failure or not accurate enough estimates are caused by unfavourable conditions, a low elevation imaging angle, and very short object-to-camera distance.

The performance of the trained model on real data was also evaluated quantitatively, using the state-of-the-art evaluation measures presented in Section 3.3, similarly to the evaluation of the laboratory model. Table 2 presents the average recall for the MSSD, MSPD, ADD and VSD metrics and the average of the average recalls for the MSSD, MSPD and VSD. All individual as well as the average recall scores exceed 94%, proving the high accuracy and precision that was achieved on unseen realistic validation data.

**Figure 20:**
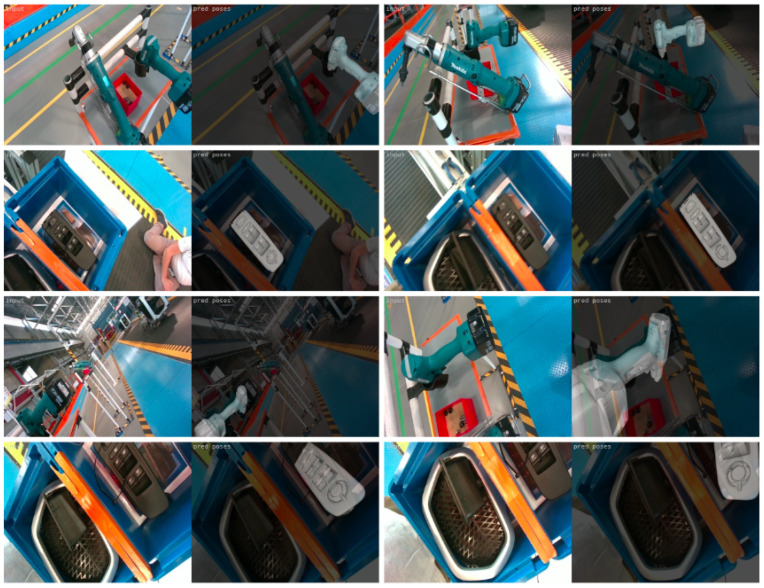
Examples of predictions on real test images. The first rows present correct object pose predictions, while the last two show incorrect ones.

The developed methodology was finally tested on a set of unseen test sequences captured in real time from the on-robot camera in the actual industrial environment. As an integrated robotic application, it managed to successfully localize the objects of interest in real conditions and in less than 0.4 s per image. Sample results on the test data are illustrated in Figure 20. The first two rows depict successful cases, while the last two, some cases of failure. As it can be observed, the method is successful in unfavourable illumination conditions, complex backgrounds with significant clutter, and even in cases of only partially occluded objects. The performance on both validation and testing data proves that, using the proper training parameters and applying online data augmentation, we avoid overfitting and achieve good generalization. However, the rare cases of failure (sampled in the last two rows) are mainly caused by extremely unfavourable conditions of clutter and occlusions or when the camera is either very close or very far from the object.In addition, training the model only on data with a complex background decreased its robustness and repeatability on less cluttered and homogeneous backgrounds, which was the advantage of the model trained on laboratory data. To overcome this and take advantage of the dynamics of both types of training data (great performance on homogeneous backgrounds for laboratory data and on complex scenes for the actual environment data), the method was also trained on a combination of laboratory and actual environment data (see Section 4.3).

### 4.3. Model Trained on Both the Laboratory and the Real Industrial Environment Data

#### 4.3.1. Datasets

To combine the advantages of the two aforementioned models in order to boost the robustness of the developed object localization method for both homogeneous, complex and unfavorable illumination scenes, it was decided to train the model also on a combination of laboratory and actual environment training data.

#### 4.3.2. Training, Validation and Testing Results

**Figure 21:**
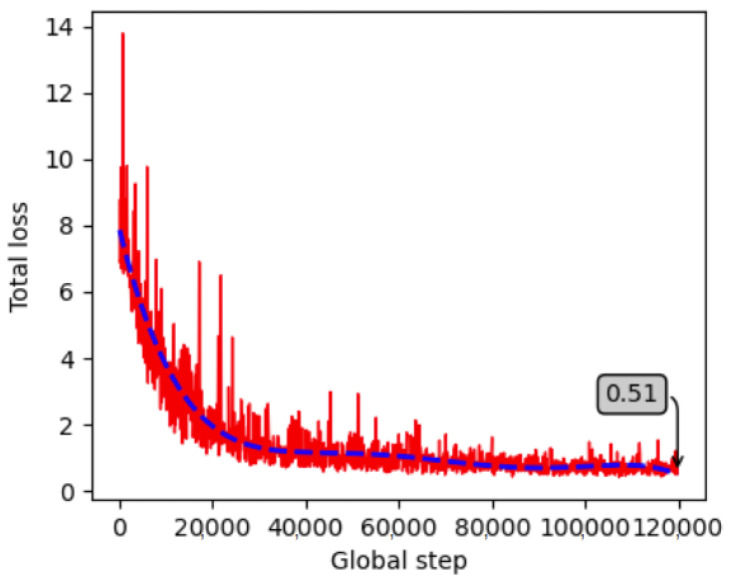
Progress of the training loss for the combined dataset (laboratory and real industrial environment).

The model in this case was trained similarly to the two models above using 774 images per object (450 from the industrial environment and 324 from the laboratory masked data). The parameters used for the final training were 120,000 steps, a 0.0001 initial learning rate, 0.9 learning power and 64 fragments per object, reaching a training loss of 0.51 (Figure 21).

As expected, the model behaved very well on all validation data. In fact, it adopted the behaviour of the first model for the validation data with the homogeneous background and the behaviour of the second model for the data with a cluttered background. This proves that overfitting was also avoided in this case, obtaining a model that is able to generalize well and cope with a create variety of scenes. The quantitative results of the validation are presented below in Table 3 such as in the previous sections.

Similarly to the training with only the real environment data, both the individual and the average recall scores exceed 94%. This demonstrates that the model is stable and able to simultaneously serve robotic applications with illumination changes and scenes that range from simpler and homogeneous to very complex and cluttered.

**Figure 22:**
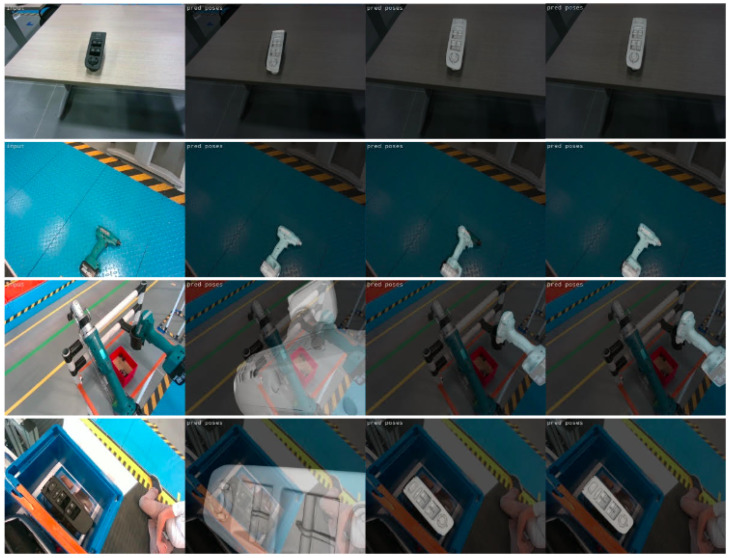
Comparison of the results of the three trained models. The first column shows the input RGB image. The rest show the results for the models trained on only laboratory, only real environment, and both laboratory and real environment data, respectively.

The performance of this model was also tested on unseen test images captured from the on-robot camera in the actual industrial environment. A comparison of the results of all the three trained models for a variety of testing data is presented in Figure 22. As seen in the second column, the model trained only on laboratory data performs quite accurately in homogeneous backgrounds (rows 1 and 2) but not in complex ones (rows 3 and 4). The model trained only on real environment data (column three) shows superior performance for complex scenes, but not always for homogeneous ones. Finally, the model trained on both laboratory and real environment data delivers correct pose estimation for all cases, verifying the potential of the method to support object-grasping in different realistic scenarios.

## 5. Conclusions

The current work presented a visual object 6D pose estimation workflow engineered to support industrial objects with challenging characteristics used in car-assembly tasks in manufacturing environments. Exploiting recent advances in 6D pose estimation based on deep learning methods, two different datasets were collected, annotated and used for model training. The first dataset consisted of images and ground truth data acquired in laboratory conditions, whereas the second dataset consisted of data collected from a real industrial environment. The pose estimation model was trained on three different combinations of data from the two datasets and was evaluated in a number of test sequences from the real car door assembly line environment. The evaluation results revealed that the model trained only on the laboratory data performs quite accurately in homogeneous backgrounds but not in complex ones. Vice versa, the model trained only on real environment data performs well for complex scenes compared to simpler scenes. Finally, the model trained with both laboratory and real environment data managed to estimate accurate object 6D poses for all cases of both simpler and complex scenes, occluded objects and illumination changes. This verifies the potential of the method to support object-grasping for car-door assembly tasks in real industrial manufacturing environments. Although the results have been proven satisfactory for the examined objects and scenes, the performance could be further improved by considering more advanced data augmentation techniques, scaling and accelerating the generation of training data based on NeRFs, while also including additional objects relevant to the application domain and that support the applicability to other manufacturing environments. Furthermore, arising from the initial assumptions of handling objects with challenging geometric and surface characteristics, that is, weak texture, symmetries, black and unfavorable environment conditions, the workflow is suitable for a number of indoor and outdoor scenarios beyond robot grasping, also for visual monitoring/tracking purposes.

## Figures and Tables

**Table 1:** Quantitative results for the evaluation of the object detection and localization model trained on laboratory data.

Recall (%)	MSSD	MSPD	ADD	VSD	Average (MSSD, MSPD, VSD)
**Masked Images**	98.3	99.1	97.1	96.0	97.8
**NON Masked Images**	50.3	54.0	44.4	48.3	50.9

**Table 2:** Quantitative results for the evaluation of the object detection and localization model trained on real environmental data.

Recall (%)	MSSD	MSPD	ADD	VSD	Average (MSSD, MSPD, VSD)
**Real Environment Images**	94.5	95.2	96.7	94.3	94.7

**Table 3:** Quantitative results for the evaluation of the object detection and localization model trained on both laboratory and real environment data.

Recall (%)	MSSD	MSPD	ADD	VSD	Average (MSSD, MSPD, VSD)
**Laboratory and Real** **Environment Images**	94.6	95.0	96.0	94.6	94.7

## Data Availability

The data used to support the findings of this study are available from the corresponding author upon request.

## References

[1] Hodaň T., Baráth D., Matas J. EPOS: Estimating 6D Pose of Objects with Symmetries. Proceedings of the IEEE Conference on Computer Vision and Pattern Recognition (CVPR).

[2] Clement F., Shah K., Pancholi D. (2019). A Review of methods for Textureless Object Recognition. arXiv.

[3] Du G., Wang K., Lian S., Zhao K. (2021). Vision-based robotic grasping from object localization, object pose estimation to grasp estimation for parallel grippers: A review. Artif. Intell. Rev..

[4] Kim S.H., Hwang Y. (2021). A Survey on Deep learning-based Methods and Datasets for Monocular 3D Object Detection. Electronics.

[5] He Z., Feng W., Zhao X., Lv Y. (2021). 6D Pose Estimation of Objects: Recent Technologies and Challenges. Appl. Sci..

[6] Sahin C., Kim T.K. Recovering 6D object pose: A review and multi-modal analysis. Proceedings of the European Conference on Computer Vision (ECCV) Workshops.

[7] Rahman M.M., Tan Y., Xue J., Lu K. (2019). Recent advances in 3D object detection in the era of deep neural networks: A survey. IEEE Trans. Image Process..

[8] Wu J., Yin D., Chen J., Wu Y., Si H., Lin K. (2020). A Survey on Monocular 3D Object Detection Algorithms Based on Deep Learning. J. Phys. Conf. Ser..

[9] Shi Y., Huang J., Xu X., Zhang Y., Xu K. (2021). StablePose: Learning 6D Object Poses from Geometrically Stable Patches. arXiv.

[10] Labbe Y., Carpentier J., Aubry M., Sivic J. CosyPose: Consistent multi-view multi-object 6D pose estimation. Proceedings of the European Conference on Computer Vision (ECCV).

[11] Jiang X., Li D., Chen H., Zheng Y., Zhao R., Wu L. Uni6D: A Unified CNN Framework without Projection Breakdown for 6D Pose Estimation. Proceedings of the IEEE/CVF Conference on Computer Vision and Pattern Recognition.

[12] Various authors (2021). Papers with Code—6D Pose Estimation Using RGB. https://paperswithcode.com/task/6d-pose-estimation.

[13] Hodaň T., Sundermeyer M., Drost B., Labbé Y., Brachmann E., Michel F., Rother C., Matas J. (2020). BOP challenge 2020 on 6D object localization. Proceedings of the European Conference on Computer Vision.

[14] Park K., Patten T., Vincze M. Pix2Pose: Pixel-wise coordinate regression of objects for 6D pose estimation. Proceedings of the IEEE/CVF International Conference on Computer Vision.

[15] Li Y., Wang G., Ji X., Xiang Y., Fox D. DeepIM: Deep iterative matching for 6D pose estimation. Proceedings of the European Conference on Computer Vision (ECCV).

[16] Tan M., Le Q. EfficientNet: Rethinking model scaling for convolutional neural networks. Proceedings of the International Conference on Machine Learning, PMLR.

[17] Dosovitskiy A., Fischer P., Ilg E., Hausser P., Hazirbas C., Golkov V., Van Der Smagt P., Cremers D., Brox T. FlowNet: Learning optical flow with convolutional networks. Proceedings of the IEEE International Conference on Computer Vision.

[18] Lepetit V., Moreno-Noguer F., Fua P. (2009). EPnP: An accurate O(n) solution to the PnP problem. Int. J. Comput. Vis..

[19] Fischler M.A., Bolles R.C. (1981). Random sample consensus: A paradigm for model fitting with applications to image analysis and automated cartography. Commun. ACM.

[20] Jin M., Li J., Zhang L. (2022). DOPE++: 6D pose estimation algorithm for weakly textured objects based on deep neural networks. PLoS ONE.

[21] He Y., Sun W., Huang H., Liu J., Fan H., Sun J. PVN3D: A Deep Point-wise 3D Keypoints Voting Network for 6DoF Pose Estimation. Proceedings of the IEEE/CVF Conference on Computer Vision and Pattern Recognition.

[22] He Y., Huang H., Fan H., Chen Q., Sun J. Ffb6d: A full flow bidirectional fusion network for 6D pose estimation. Proceedings of the IEEE/CVF Conference on Computer Vision and Pattern Recognition.

[23] He Y., Wang Y., Fan H., Sun J., Chen Q. FS6D: Few-Shot 6D Pose Estimation of Novel Objects. Proceedings of the IEEE/CVF Conference on Computer Vision and Pattern Recognition.

[24] Cao T., Luo F., Fu Y., Zhang W., Zheng S., Xiao C. DGECN: A Depth-Guided Edge Convolutional Network for End-to-End 6D Pose Estimation. Proceedings of the IEEE/CVF Conference on Computer Vision and Pattern Recognition.

[25] He Z., Zhang L. Multi-adversarial faster-RCNN for unrestricted object detection. Proceedings of the IEEE/CVF International Conference on Computer Vision.

[26] Li F., Yu H., Shugurov I., Busam B., Yang S., Ilic S. (2022). NeRF-Pose: A First-Reconstruct-Then-Regress Approach for Weakly-supervised 6D Object Pose Estimation. arXiv.

[27] Hinterstoisser S., Lepetit V., Ilic S., Holzer S., Bradski G., Konolige K., Navab N. (2012). Model based training, detection and pose estimation of texture-less 3D objects in heavily cluttered scenes. Proceedings of the Asian Conference on Computer Vision.

[28] Brachmann E., Krull A., Michel F., Gumhold S., Shotton J., Rother C. (2014). Learning 6D object pose estimation using 3D object coordinates. Proceedings of the European Conference on Computer Vision.

[29] Kaskman R., Zakharov S., Shugurov I., Ilic S. Homebreweddb: RGB-D dataset for 6D pose estimation of 3D objects. Proceedings of the IEEE/CVF International Conference on Computer Vision Workshops.

[30] Rennie C., Shome R., Bekris K.E., De Souza A.F. (2016). A dataset for improved RGBD-based object detection and pose estimation for warehouse pick-and-place. IEEE Robot. Autom. Lett..

[31] Tejani A., Tang D., Kouskouridas R., Kim T.K. (2014). Latent-class Hough forests for 3D object detection and pose estimation. Proceedings of the European Conference on Computer Vision.

[32] Xiang Y., Schmidt T., Narayanan V., Fox D. (2017). PoseCNN: A convolutional neural network for 6D object pose estimation in cluttered scenes. arXiv.

[33] Hodan T., Haluza P., Obdržálek Š., Matas J., Lourakis M., Zabulis X. (2017). T-LESS: An RGB-D dataset for 6D pose estimation of texture-less objects. Proceedings of the 2017 IEEE Winter Conference on Applications of Computer Vision (WACV).

[34] Drost B., Ulrich M., Bergmann P., Hartinger P., Steger C. Introducing MVTec ITODD—A dataset for 3D object recognition in industry. Proceedings of the IEEE International Conference on Computer Vision Workshops.

[35] Doumanoglou A., Kouskouridas R., Malassiotis S., Kim T.K. Recovering 6D object pose and predicting next-best-view in the crowd. Proceedings of the IEEE Conference on Computer Vision and Pattern Recognition.

[36] Chang A.X., Funkhouser T., Guibas L., Hanrahan P., Huang Q., Li Z., Savarese S., Savva M., Song S., Su H. (2015). Shapenet: An information-rich 3D model repository. arXiv.

[37] Byambaa M., Koutaki G., Choimaa L. (2022). 6D Pose Estimation of Transparent Objects Using Synthetic Data. Proceedings of the International Workshop on Frontiers of Computer Vision.

[38] Hodan T., Michel F., Brachmann E., Kehl W., GlentBuch A., Kraft D., Drost B., Vidal J., Ihrke S., Zabulis X. BOP: Benchmark for 6D object pose estimation. Proceedings of the European Conference on Computer Vision (ECCV).

[39] Chen L.C., Zhu Y., Papandreou G., Schroff F., Adam H. encoder–decoder with atrous separable convolution for semantic image segmentation. Proceedings of the European Conference on Computer Vision (ECCV).

[40] Huber P.J. (1992). Robust estimation of a location parameter. Breakthroughs in Statistics.

[41] Barath D., Matas J. Progressive-x: Efficient, anytime, multi-model fitting algorithm. Proceedings of the IEEE/CVF international Conference on Computer Vision.

[42] Barath D., Matas J. Graph-cut RANSAC. Proceedings of the IEEE Conference on Computer Vision and Pattern Recognition.

[43] Kneip L., Scaramuzza D., Siegwart R. (2011). A novel parametrization of the perspective-three-point problem for a direct computation of absolute camera position and orientation. Proceedings of the CVPR 2011.

[44] Moré J.J. (1978). The Levenberg-Marquardt algorithm: Implementation and theory. Numerical Analysis.

[45] Hodaň T., Matas J., Obdržálek Š. (2016). On evaluation of 6D object pose estimation. Proceedings of the European Conference on Computer Vision.

[46] Ronneberger O., Fischer P., Brox T. (2015). U-net: Convolutional networks for biomedical image segmentation. Proceedings of the International Conference on Medical Image Computing and Computer-Assisted Intervention.

[47] Cignoni P., Callieri M., Corsini M., Dellepiane M., Ganovelli F., Ranzuglia G. Meshlab: An open-source mesh processing tool. Proceedings of the Eurographics Italian Chapter Conference.

[48] Intel RealSense Depth Camera D455. https://www.intelrealsense.com/depth-camera-d455/.

[49] Garrido-Jurado S., Muñoz Salinas R., Madrid-Cuevas F., Marín-Jiménez M. (2014). Automatic Generation and Detection of Highly Reliable Fiducial Markers under Occlusion. Pattern Recognit..

[50] Long J., Shelhamer E., Darrell T. Fully convolutional networks for semantic segmentation. Proceedings of the IEEE Conference on Computer Vision and Pattern Recognition.

[51] Chen L.C., Papandreou G., Schroff F., Adam H. (2017). Rethinking atrous convolution for semantic image segmentation. arXiv.

[52] Badrinarayanan V., Kendall A., Cipolla R. (2017). Segnet: A deep convolutional encoder–decoder architecture for image segmentation. IEEE Trans. Pattern Anal. Mach. Intell..

[53] Lourakis M. Posest: A C/C++ Library for Robust 6DoF Pose Estimation from 3D-2D Correspondences. https://users.ics.forth.gr/~lourakis/posest/.

[54] Lourakis M., Zabulis X. (2013). Model-based pose estimation for rigid objects. Proceedings of the International Conference on Computer Vision Systems.

[55] Chollet F. Xception: Deep learning with depthwise separable convolutions. Proceedings of the IEEE Conference on Computer Vision and Pattern Recognition.

[56] Deng J., Dong W., Socher R., Li L.J., Li K., Fei-Fei L. (2009). Imagenet: A large-scale hierarchical image database. Proceedings of the 2009 IEEE Conference on Computer Vision and Pattern Recognition.

[57] Lin T.Y., Maire M., Belongie S., Hays J., Perona P., Ramanan D., Dollár P., Zitnick C.L. (2014). Microsoft COCO: Common objects in context. Proceedings of the European Conference on Computer Vision.

[58] Hinterstoisser S., Lepetit V., Wohlhart P., Konolige K. On pre-trained image features and synthetic images for deep learning. Proceedings of the European Conference on Computer Vision (ECCV) Workshops.

[59] Blume F. 6DPAT. https://github.com/florianblume/6d-pat.

